# The effects of stress on surgical performance: a systematic review

**DOI:** 10.1007/s00464-024-11389-3

**Published:** 2024-12-03

**Authors:** Adam Tam, Samuel Bateman, Gavin Buckingham, Mark Wilson, G. J. Melendez-Torres, Sam Vine, James Clark

**Affiliations:** 1 Department of Surgery, Royal Cornwall Hospitals NHS Trust, Truro, UK; 2 Public Health and Sport Sciences, University of Exeter, Exeter, UK; 3 University of Exeter Medical School, Exeter, UK

**Keywords:** Acute stress, Surgical performance, Technical skills, Non-technical skills

## Abstract

**Introduction:**

Acute stress, the psychological response to short-term challenging stimuli, is frequently encountered in the high-pressure environment of the operating theatre. Surgeon stress is associated with deterioration in surgical performance, surgical team working and compromised patient safety. Given these concerns, the aim of this review was to understand the impact of acute stress on surgical performance in technical and non-technical domains as well as patient outcomes.

**Methods:**

A systematic review was conducted following PRISMA guidelines. Electronic databases were searched for studies examining acute stress in medical professionals during real or simulated surgical procedures that reported performance outcomes in technical and non-technical skills. Risk of bias assessment was conducted using appropriate tools for each study design.

**Results:**

Out of 1445 identified studies, 19 met the inclusion criteria. In simulated environments, acute stress consistently led to impairment in both technical and non-technical skills during surgical procedures. Technical skill deterioration included higher procedural error rates, longer task completion times and diminished instrument handling. Non-technical skills, such as teamwork and communication, also were impaired under stress conditions. Real-world studies echoed these findings, demonstrating acute stress resulting in higher error rates and impaired non-technical skills. No studies identified surgeon stress as having a causal relationship with patient outcomes.

**Conclusion:**

Acute stress significantly impacts both technical and non-technical skills during surgical procedures, impairing performance in simulated and real-world surgical environments. Despite the growing understanding of the detrimental effects of stress, gaps remain in comprehensively assessing its impact on patient outcomes. Further research is warranted to develop reliable stress measurement methods applicable in surgical settings and explore effective stress management strategies.

**Supplementary Information:**

The online version contains supplementary material available at 10.1007/s00464-024-11389-3.

The operating theatre is a high-pressure environment, where time-critical decision making and immediate consequence can induce surgeon stress. The American Psychological Association defines stress as “the physiological or psychological response to internal or external stressors” [1]⁠⁠. Stress can also be thought of as a condition that results in a state of arousal, in preparation for a response or adaption [2]⁠⁠. From a physiological perspective, the perception of a stressor involves a vast array of neuronal networks [3, 4]⁠⁠⁠. There differences in the parthway utilised for processing between physical and psychological stimuli, with physical stressors being processed largely in the hypothalamic and brainstem regions and the latter having greater involvement of higher cortical regions [3]⁠.

Stress can also be defined using different classifiers such as the source, timing or psychological experience of the stressor. Psychological stress can be experienced from acute (unexpected intraoperative haemorrhage) or chronic events (general life stressors and burnout). Within health research, acute stress is generally defined as short-term exposure to external stressors, whilst internal stressors are typically associated with chronic stress [5]⁠. There is some suggestion that the response to the external stressor may be modulated by the internal environment [5, 6]⁠. In reality, both sources co-exist, jointly contributing to the psychophysiological response [5, 6]⁠.

Acute stress can result from multiple sources within the complex environment of the operating theatre. Arora et al. demonstrated high levels of surgeon stress from several factors that could compromise patient safety: patient problems, technical problems as well as equipment and team-work issues [7]⁠. Attempts have been made to clarify the relationship between these factors, such as the Surgical Stress Effects Framework [8]⁠. This model identifies how individual and team characteristics, influenced by organisational and environmental conditions, dynamically interact with job demands and resources. These summate in a stress response affecting both individual and team performance. This performance impacts patient outcome with various factors posing immediate or latent safety threats, errors and adverse events [8]⁠. It is therefore clear that there is a complex relationship between stress and surgical performance.

There are also longer-term effects with exposure to acute stress, with evidence suggesting prolonged exposure contributing to the development of mental health disorders [9]⁠⁠. This may partially explain the high prevalence of mental health disorders in healthcare workers, with 10–20% of doctors experiencing depression during their career and suicide being a disproportionate cause of death amongst doctors relative to the general population [10]⁠⁠. Stress is also an important factor in workforce sustainability with high prevalence of burnout amongst surgeons contributing to work place shortages [11]⁠⁠. Surgeons appear to understand the negative effects of stress, although less insightful views of stress as a “sign of failure” that should be “tolerated” have also been identified [12]⁠. Despite perceptions of resilience, surgeons are not exempt from the consequences of stress and the interaction between acute and chronic stressors within the operating environment is still poorly understood.

Alongside the mental health of the surgeon, there is also evidence to suggest that acute stress has a negative impact surgical performance and thus patient outcomes [13]⁠**.** Superior technical skills have been shown to have a direct impact on both short-term and long-term patient outcomes [14, 15]⁠. This has been demonstrated in technical domains such as procedural error as well as non-technical domain skills such as communication and teamwork [8, 13, 14]⁠⁠⁠. The effect of stress is not only limited to the operating surgeon themselves, with emotional “contagion” transferring stressed emotional states to other surgical team members [8, 12]⁠. Patients are also affected by the resultant deterioration in non-technical skills, with a third of communication errors resulting in errors endangering patient safety [16]⁠. These negative effects can also occur in the chronic setting. In a systematic review, surgeons reporting burnout were shown to have a 2.5-fold increased risk of making medical errors [11]⁠.

Although chronic stress is important, the acute stress that is experienced during a surgical procedure is a prime target for the utilisation of training and technology within the operating theatre. Advances in simulation technology, including virtual and augmented reality, are increasingly being utilised to assess surgeons responses to stress in a safe environment. Although previous reviews on surgical stress have, by and large, not specifically looked at performance in the context of understanding stress within surgery [13, 14, 17, 18]⁠. Furthermore, none of these reviews attempted to define the effect of stress on patient outcomes. Thus, the primary objective of the current article is an up to date review on the effects of acute psychological stress on both technical and non-technical surgical skills in both simulated environments and in real-world surgery. The secondary objective aims to define the literature on how stress in operating surgeons relates to patient outcomes.

## Methods

A systematic review was performed in accordance with the Preferred Reporting Items for Systematic reviews and Meta-Analyses (PRISMA) [19]⁠⁠. The review was pre-registered prior to searching (CRD42024529018). An electronic search of databases including PsycINFO, Ovid MEDLINE, EMBASE, Cochrane Database was performed on 19/03/2024. Screening was performed by two reviewers independently and a separate adjudicator was available in the event of inter-rater differences. Cognitive load and anxiety, common synonyms of stress, were also used as search terms given the variable definitions of stress used in the literature. Acute stress was defined in this review as a short-term direct or indirect exposure to a stressor. The search strategy is detailed in Appendix 1. The search was repeated prior to submission for publication (02/06/2024) to ensure no recent publications were omitted.

Inclusion criteria are summarised in Table 1. Studies without defined comparators were included if stress was demonstrated during the exposure period. Comparators could also include situations where surgeons were not under stress or a recognized lower stress situation (e.g., assistant vs primary operator, low vs high risk procedure or elective vs emergency surgery). However studies that compared stress between two methods of surgery (for example robotic versus laparoscopic surgery) were excluded as a causality between stress and outcome cannot be ascertained. Measurements of stress could include subjective measures such as questionnaires and objective measures such as validated physiological measurements (for example heart rate variability). Retrospective measurement of stress was included if the exposure to stress was in the peri-procedural period.

**Table 1:** Inclusion and exclusion criteria

Inclusion criteria	Exclusion criteria
Any grade of surgeon or physician (including medical students) performing a real or simulated surgical procedure both in physical or extended reality	Non-surgeon populations
Procedures from all surgical specialties	Studies in mixed populations where outcomes of surgeons could not be defined separately
Simulated or real-world environments	Real-world non-human surgery
Acute stress during the peri-procedural period	Chronic stress, mental health, or longer-term stress
Quantitative research including observational studies and experimental studies. Mixed methods studies are included if they meet inclusion criteria and have suitable results for quantitative synthesis	Qualitative studies

The main outcome was surgical performance in either technical or non-technical domains during the procedure. Technical domains include but were not limited to outcomes such as dexterity, procedural length and blood loss as well as validated scoring systems such as the Observed Structured Assessment of Technical Skills (OSATS). Non-technical domains include but were not limited to outcomes such as verbal communication, situational awareness or other forms of validated non-technical skills testing. Studies that did not contain a measure of surgeon performance or did not measure this at the time of the procedure were excluded.

After screening, the following data were extracted: Publication details, study data, participant data, data related to acute stress exposure; including timing, nature of stress and the validated or non-validated measurement tools used to measure either physiological or psychological reaction to stress and the use of self-report or objective metrics; outcomes in technical and non-technical domains as well as patient outcomes. Results were synthesised qualitatively through descriptive and thematic analysis covering technical, non-technical skills and patient outcome.

Risk of bias was assessed using the Newcastle–Ottawa Scale for observational studies, the ROBINS-I score for non-randomised experimental studies and the Rob2 score for randomised controlled trials [20–22]⁠.

## Results

**Fig. 1:**
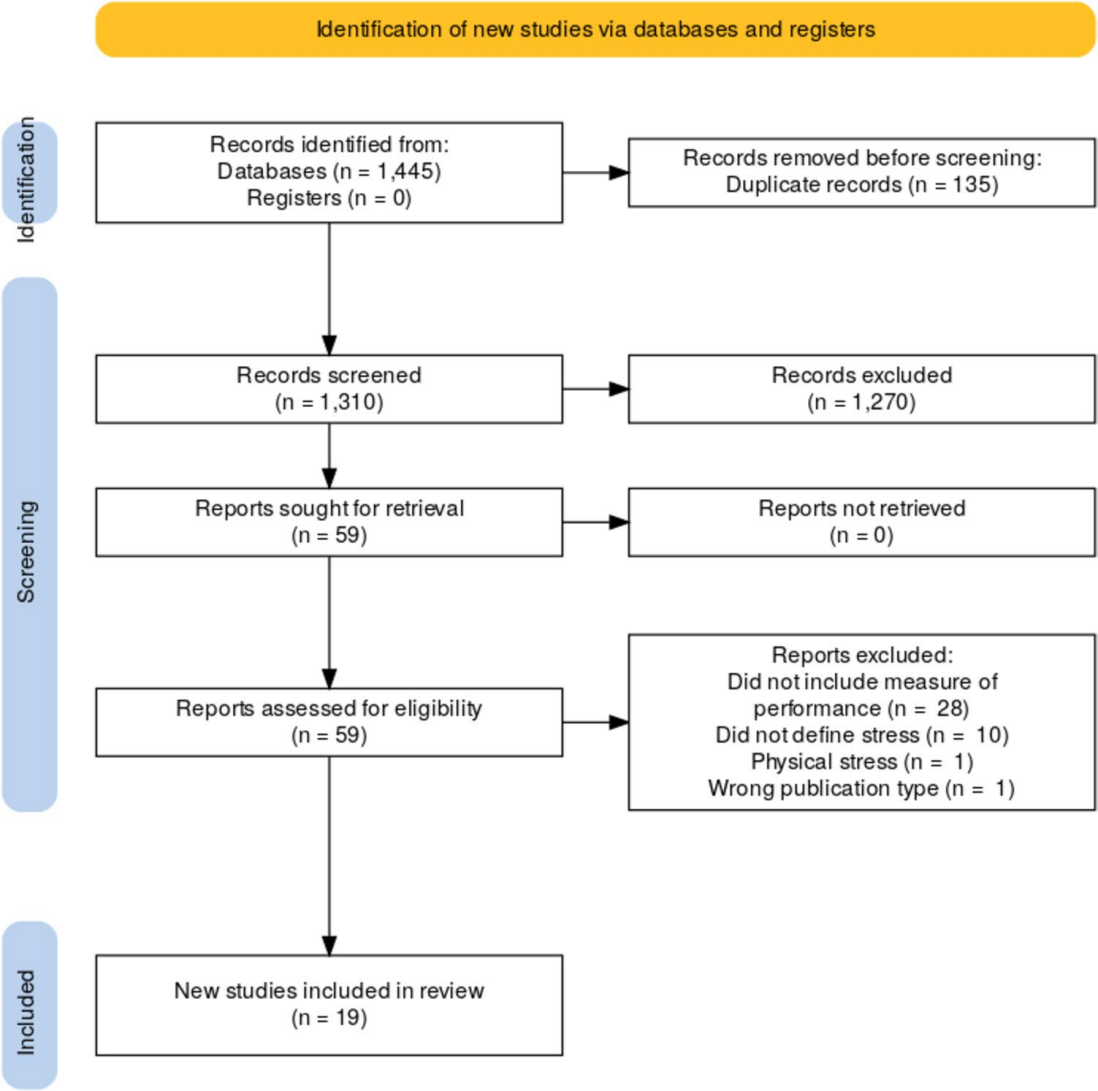
PRISMA flow diagram of included studies

In total, 1445 studies were identified and screened by two separate reviewers (see Fig. 1) with substantial inter-rater agreement (Cohens κ = 0.75, SE = 0.09, CI 0.58–0.92). A total of 19 articles fulfilled the inclusion criteria, consisting of ten non-randomised, prospective experimental studies, five randomised controlled trials and four prospective observational trials. Study summaries are shown in Table 2. Twelve studies involved direct exposure to a stressful stimulus (Five simulated patient-related crises, three additional cognitive loading, one exposure to different music, one exposure to different ambient temperature, one exposure to visual stimuli, one simulated procedure on a live animal). Only four studies were crossover trials [23–26]⁠. Risk of bias was low to moderate for most studies, with six studies rated low risk, twelve studies medium risk and one high risk of bias.

**Table 2:** Summary of included studies

Author	Year	Study type	Type of surgeon	Surgeon experience	Live or simulated procedure	Participants	Surgery types	Stressor
Keenlyside et al. [24]	2023	RCT	General	Medical Students	Simulated	30	Simulated laparoscopic peg task	Exposure to validated positive, neutral and negative image stimuli (Open Affective Standardised Image Set)
Zheng et al. [25]	2022	RCT	General	Medical Students	Simulated	30	Simulated laparoscopic peg task	Progressive deterioration in simulated patient physiology and cardiac arrest, observer provided auditory distraction as well as asked participant to complete task more quickly
Anton et al. [35]	2021	Prospective observational	Neurosurgery, cardiothoracics, other	Fellows, Residents	Live	15	Not described	Surgical cases: not comparative
Van Houwelingen et al. [36]	2020	Prospective observational	General	Surgeons (novice, expert)	Live	8	Open and Laparoscopic general surgery including oesophagectomy and cholecystectomy	Surgical cases: not comparative
Grantcharov et al. [34]	2019	Prospective observational	General	Surgeon	Live	1	Gastric bypass, sleeve gastrectomy, peroral endoscopic myotomy	Surgical cases: not comparative
Bakhsh et al. [33]	2019	Prospective Experimental	Vascular	Residents	Simulated	10	Thoracic Endovascular Anuerysm Repair (TEVAR)	Simulated Crisis (not described)
Sexton et al. [37]	2018	Prospective observational	Urology	Surgeon	Live	3	Robot assisted prostatectomy	Surgical cases: not comparative
Stefanidis et al. [39]	2017	RCT	General	Medical Students	Simulated	46	Laparoscopic peg task and suturing followed by retention task in a live animal model	Live animal procedure
Crewther et al. [40]	2016	Prospective Experimental	General	Medical Students	Simulated	12	Simulated laparoscopic suturing	Simulated cases: Not comparative
Chung et al. [22]	2016	Prospective Experimental	General	Medical Students	Simulated	53	Simulated robotic tasks (energy dissection, suture sponge)	3 different auditory environments (classical, neutral, death metal)
Louridas et al. [30]	2015	RCT	General	Residents	Simulated	20	Laparoscopic jejunostomy	Not comparative: Crisis scenario (anaphylaxis in simulated patient mid-procedure)
Berg et al. [20]	2012	Prospective Experimental	General	Residents	Simulated	21	Laparoscopic peg transfer and knot tie	Change in theatre ambient temperature
Poolton et al. [21]	2011	Prospective Experimental	General	Medical Students	Simulated	30	Simulated laparoscopic peg task	Dual-tasking, evaluative threat, time pressure
Wetzel et al. [28]	2011	RCT	Vascular	Residents	Simulated	16	Carotid Endarterectomy	Simulated stressful intra-operative events (not described)
Arora et al. [26]	2011	Prospective Experimental	General	Residents	Simulated	18	MIST-VR simulator tasks (transfer, diathermy and withdraw and insert)	Simulated cases: Not comparative
Shuetz et al. [27]	2008	Prospective Experimental	General	Surgeons	Simulated	18	LapSim laparoscopic cholecystectomy	Simulated cases: Not comparative
Moorthy et al. [31]	2006	Prospective Experimental	General	Surgical trainees	Simulated	20	Simulated bleeding crisis	Unexpected bleeding within scenario
Hassan et al. [29]	2006	Prospective Experimental	General	Medical Students and Junior residents	Simulated	24	Larparoscopic task: diathermy task	Task set to difficult setting and observer giving negative feedback
Moorthy et al. [23]	2003	Prospective Experimental	General	Surgeon	Simulated	13	Laparoscopic tasks under 5 conditions	Dual-task, background noise, as quickly as possible, all three stressors combined and quiet conditions

### The effect of acute stress during surgical procedures in simulated environments

Fifteen studies were identified in simulated environments (Table 3).

**Table 3:** Effect of acute stress on performance in simulated environments

Author	Year	Stress measurement	Timing of stress measurement	Measurement tool	Technical	Non-technical	Patient outcome	Critique	Risk of Bias (Newcastle-Ottowa)	Author conclusions
Anton et al. Am J Surg	2021	STAI-6, SURG-TLX	Intra-operatively, post-operatively (questionnaires)	Non-Technical Skills for Surgeons (NOTSS)	Not measured	Stress had negative relationship with situational awareness, decision making, mental workload, situational stress, distractions. Mental workload and situational stress negatively correlated with considering options. Overall higher stress and workload associated with lower non-technical skills regardless of experience level	Not measured	Post-procedure and self-reported measurement. Small sample size. Observer effect	NOS = 6 (moderate)	Surgeons’ stress and workload negatively affected their non-technical skills. Further, unfamiliarity contributed to surgeon stress
Van Houwelingen et al. Surg Endosc	2020	Heat Flux, METs, GSR	Intra-operatively	Task engagement (Metabolic Equivalent of Tasks, MET)	Not measured	Stress level and task engagement are affected differently between long and short procedures. Task engagement increased throughout procedure regardless of length. Surgical flow disruption is taxing on surgeons mental resources as a function of the length and nature of disruptions	Not measured	Pilot study, full detail only described for 2 cases	NOS = 6 (moderate)	Interruptions and disruptions affect novice and expert surgeons differently. Surgical flow disruption appears to be taxing on the surgeons’ mental, emotional and physiological resources; as a function of the length and nature of the disruptions
Grantcharov et al. BJS Open	2019	HRV (SDNN, RMSSD)	Intra-operatively	Generic Error Rating Tool (GERT)	Higher error rate in the highest HRV quantiles than the lowest quantiles using both SDNN and RMSSD over a 1,2 and 5-min time frames	Not measured	Not measured	Single surgeon	NOS = 3 (high)	There is an association between measures of acute mental stress and worse technical surgical performance,
Sexton et al. BMJ Quality and Safety	2018	NASA-TLX	Post-operatively (questionnaires)	Number of requests (verbal and non-verbal), anticipation and inconvenience	Not measured	Surgeon cognitive load (NASA-TLX) significantly correlated with anticipation ratio, percent of non-verbal requests and total request duration	Not defined between higher and lower stress	Observational, some parts of surgery missed due to video issues	NOS = 6 (moderate)	Anticipation and active engagement by the surgical team resulted in shorter operative time, and higher familiarity scores were associated with fewer inconveniences. Less anticipation and non-verbal requests were also associated with lower cognitive load for the console surgeon

#### Technical skills

In a simulated environment, ten prospective experimental studies and five randomized trials were identified with low to medium risk of bias. All studies demonstrated deterioration of technical skills in response to acute stress. Six studies demonstrated the negative impact of stressors on operator kinematics [24, 26–30]⁠. Increased instrument path length, velocity, acceleration and jerk as well as deterioration in usage metrics such as economy of motion, economy of volume and smoothness were also demonstrated during stressed conditions [24, 28, 29]⁠. Deterioration in scores for tissue handling, instrument handling as well as higher procedural error rates were demonstrated in six studies [26, 29, 31–34]⁠. Zheng et al. demonstrated a linear relationship between instrument velocity, acceleration, jerk as well as path length and increasing levels of stress, with a corresponding reduction of economy of motion under increasing stress [28]⁠. Moorthy et al. reported an increase in time to diagnose and control unexpected bleeding in novices who reported higher stress scores [34]⁠⁠.

Stress was demonstrated to increase the time taken to perform the procedures as well as a general decline in operative performance [24, 27, 29, 35]⁠. Despite the heterogeneity of the simulated procedures involved, from peg transfer to more realistic femoral vein exposure, all studies described a decrease in global technical skills, more rapid and less economical movements in response to stress. Four studies compared non-stress performance against simulated patient crises, although only two of these validated the stress event with subjective or objective markers of stress [28, 33, 34, 36]⁠⁠. Both realistic and non-realistic stressors resulted in similar impairments of performance, suggesting that these laboratory-based findings could have real-world implications. However, the confidence of these findings is weakened given the heterogeneity in study design and mixed use of comparative and non-comparative stress exposure.

Three studies did not demonstrate deteriorations in performance. Bahksh et al. did not demonstrate any global difference in technical performance in simulated carotid endarterectomy, although increased sympathetic tone (a physiological maker of stress) was associated with poorer technical performance [36]⁠. The two other studies that did not demonstrate a negative effect of stress used external sensory manipulations which the authors acknowledged was likely insufficiently stressful enough to provoke a response [23, 25]⁠. Chung et al. did not demonstrate any significant difference in performance during exposure to three different auditory stimuli and Berg et al. did not demonstrate any difference in technical performance between groups exposed to two different ambient temperatures although they did note increased physical demand and distraction [23, 25]⁠.

#### Non-technical skills

Two randomised trials of low risk of bias and a single prospective experimental study of moderate risk of bias demonstrated non-technical skill stress effects [32, 34, 35]⁠. In a comparison between a mental practice and conventional practice group, a statistically significant decrease in teamwork scores (OSATS) was demonstrated in a group with higher stress reported during a simulated carotid endarterectomy [35]⁠. In a simulated bleeding crisis, Moorthy et al. did not demonstrate any overall difference in human factors skills although noted a higher number of communications to the surgical team from the expert group who reported lower stress [34]⁠. Louridas et al. compared non-technical skills in jejunostomy formation with a simulated mid-procedural crisis, however, no deterioration in non-technical skills was demonstrated [32]⁠. However the authors acknowledged that the stress-inducing scenario may have not been adequate and relatively insensitive metrics (averaged heart rate and blood pressure) were used to measure stress [32]⁠. The overall lack of significant findings may be a result of the large array of tools used to measure non-technical skills alongside large heterogeneity of study design.

### The effect of acute stress during real-world surgical procedures

Four prospective observational studies were performed in real-world operating theatres. All studies did not have a comparative measure between stress and non-stressed states but demonstrated higher levels of stress affecting performance (Table 4).

**Table 4:** Effect of acute stress on performance in simulated environments

Author	Year	Stress measurement technique	Timing of stress measurement	Technical skill measurement tool	Impact on technical skills	Impact on non-technical skills	Risk of Bias (1: Rob2, 2: ROBINS-I)	Author conclusions
Keenlyside et al. [24]	2023	Not measured	Not described	Time taken to complete task, distance travelled by instrument tips, handedness, average speed of instrument tips, average motion smoothness	No statistical difference in performance seen between stimuli groups. Rapid decrease in time taken for task completion in the group exposed to positive stimuli. Also increase in instrument speed and motion smoothness. Decrease in instrument speed in negative stimulus group	Not measured	Low ^1^	The effect of emotive visual stimuli on surgical performance is complex
Zheng et al. [25]	2022	Not measured	Not described	Modified Observed Structured Assessment Tool, Velocity, acceleration, jerk, path length, economy of volume of instrument tip	Stressed group had statistically significant higher velocity, acceleration and jerk in both hands. Stressed group had poorer mOSATS scores (respect for tissue, instrument handling and total score). Greater stress intensity resulted in greater velocity, acceleration, jerk, path length and reduced economy of volume. Stressed group also had deterioration of performance in terms of mOSATS domains of respect for tissues, instrument handling, total score	Not measured	Low ^1^	The clinical stressors had a negative effect on surgical performance. Kinematic metrics are negatively impacted by stress conditions for both the dominant and non-dominant hands. Stressed group’s movement faster and less smooth. Control group had better performance in treating the tissue, handling and moving with the instruments
Bakhsh et al. [33]	2019	HR, R-Ri, LF/HF, NASA-TLX^*^	Intra-operatively, post-operatively (questionnaires)	Global Rating Scale of Endovascular Performance (GRS-E)	No global difference in technical performance, individual increased sympathetic tone was associated with poorer technical performance, however the difference was not significant	Not measured	Low ^2^	A discrete, measurable increase in stress is experienced by surgeons during high fidelity endovascular simulation and differentially affects junior surgeons
Stefanidis et al. [39]	2017	STAI-6, HR^*^	Intra-operatively, post-operatively (questionnaires)	Objective score based on completion within cutoff time, task completion time, accuracy error, knot security error	Mental skills practice group had lower stress in transfer and retention tests (not statistically significant in retention task) although average HR was higher compared to control. Suturing performance increased in retention task in the mental skills group	Not measured	Low ^1^	The mental skills practice implemented in this study effectively enhanced participants’ mental skill use, laparoscopic performance and reduced cognitive stress
Crewther et al. [40]	2016	STAI-6, salivary cortisol, HR, HRV, NASA-TLX, f-NIRS^*^	Intra-operatively, post-operatively (questionnaires)	Fundamentals of Laparoscopic Surgery scoring system consisting of efficiency (time), precision (errors)	Stress significantly affected task performance. Skill performance showed improvement with lower stress and workload reported at later stages. HR data exhibited lower values in the middle and post sessions compared to baseline, suggesting reduced stress. Moreover, stress and workload measures were negatively correlated with both absolute skill performance and its percentage changes over time	Not measured	Moderate ^2^	A 3-week laparoscopic training program promoted stress-related adaptations supporting the acquisition of new surgical skills and many outcomes were retained after a 4-week period without further training
Chung et al. [22]	2016	MAP^*^	Intra-operatively	Simulator scoring	Classical music had a significant effect on reducing MAP during both the Energy Dissection and Suture Sponge tasks compared to the no music and death metal music. While there were no significant differences in simulator scores among the three groups, the physiological and perceived effects suggest that stress reduction elicited from music type	Not measured	Moderate ^2^	Listening to classical music during surgical training of novice robotic surgeons was associated with a reduction in stress level as evidenced by a lowering of blood pressure. This did not impact performance scores however
Louridas et al. [30]	2015	STAI-6, HR, BP^*^	Intra-operatively, post-operatively (questionnaires)	Objective Structured Assessment Tool, bariatric OSATS, NOTSS	Mental practice arm had improved technical performance (OSATS, BOSATS) overall in crisis scenario. Seven of ten trainees improved their technical performance during the crisis scenario. Mental imagery ability improved significantly following mental practice training, but not in the conventional group. No differences in objective or subjective stress levels or non-technical skills were evident	No difference in non-technical skills between groups	Low ^1^	Mental practice improves technical performance for advanced laparoscopic tasks in the simulated operating room, and allows trainees to maintain or improve their performance despite added stress
Berg et al. [20]	2012	SURG-TLX^*^	Post-operatively	FLS-based scoring system including time taken to complete task and errors	Increase in operating room ambient temperature increased perceptions on physical demand and distraction however no difference in technical performance	Not measured	Moderate ^2^	Surgeons do report increased perceptions of distraction and physical demand from increasing ambient temperature although this does not decrease technical performance in short operative tasks
Poolton et al. [21]	2011	STAI-6, HR^*^	Intra-operatively, post-operatively (questionnaires)	FLS scoring including completion time, path length	Time pressure condition perceived as significantly more stressful than control or evaluation conditions. Correlation analyses showed weak associations between STAI and heart rate measures, particularly in the multitasking condition. Notably, completion time and path length analyses demonstrated significant effects of stress, with the multitasking condition resulting in slower completion times and longer path lengths compared to other conditions	Not measured	Moderate ^2^	Recommended measures of stress levels do not necessarily reflect the demands of an operative task, highlighting the need to understand better the mechanisms that influence performance in surgery
Wetzel et al. [28]	2011	STAI-6, HR, HRV coefficient, salivary cortisol^*^	Intra-operatively, post-operatively (questionnaires)	OSATS, Observational Teamwork Assessment for Surgery (OTAS), Surgical Decision making, Number of surgical coping strategies	Tendency for lower technical surgical skills (OSATS) in control group who demonstrated increased stress	Mental practice improved OTAS performance. No significant changes in control group. Lower stress in the mental practice group improved team working skills	Low ^1^	Stress coping, mental rehearsal and relaxation training had beneficial effects on coping, stress, and nontechnical skills during simulated surgery
Arora et al. [26]	2010	STAI, Imperial Stress Assessment Tool^*^	Intra-operatively, post-operatively (questionnaires)	Dexterity parameters (path length, time taken, number of errors)	Stress correlated with economy of motion and number of errors. Objective stress correlated significantly with time taken, economy of motion and number of errors	Not measured	Moderate ^2^	Stress impairs surgical performance on a simulator. Higher stress levels correlate with increased time required for task completion, poorer economy of motion and increased errors
Shuetz et al. [27]	2008	Skin resistance	Intra-operatively	Manual surgical skills (path length, angular path), intraoperative failures (blood loss, missing clips, vessels ripped)	Laparoscopic extensions of movement were larger in stress condition without recovery as well as cumulative deviation of degree from the optimal path compared with those showing no stress reaction or recovery from stress reaction. However fewer intra-operative failures in stress group	Not measured	Moderate ^2^	The mental load of the laparoscopic surgeon might be highly optimized by continuous activity of the sympathetic nervous system. The question of what extent or quality of stress produces adverse effects remains unclear
Moorthy et al. [31]	2006	Not measured	-	Generic Rating Scale for Technical Ability to Control Bleeding, blood loss, NOTECHs, Communications count, utterance frequency, time measures of crisis management	Novices had lower technical skill. Significantly greater blood loss in novice group. Significant differences in Time to diagnose bleeding, achieve control and laceration closure. Novices more likely to perform actions detrimental to patient safety (blind application and inappropriate use of traumatic clamps as well as more likely to focus on closing the laceration at the cost of blood loss)	No differences between junior and senior groups aside from higher number of communications per minute in the senior group	Moderate ^2^	Variability in performance both in between and within the 2 groups of junior and senior surgical trainees
Hassan et al. [29]	2006	Not measured	-	time to complete the task, number of errors, economy of motion, angular path, instrument path length	Stress management strategies significantly impact laparoscopic simulator performance. Negative stress coping correlates with longer task completion time at the ‘easy’ level, while during training, it does not correlate with performance metrics. However, at the ‘difficult’ level, negative coping correlates with longer task completion time, more errors, and less efficient motion. Similarly, distraction correlates with poorer performance in the final task for time, errors and motion	Not measured	Moderate ^2^	Ineffective stress-coping strategies correlate with poor virtual laparoscopic performance. The need for effective intra-operative stress-coping strategies is evident
Moorthy et al. [23]	2003	Not measured	-	Imperial College Surgical Assessment Device (measuring manual dexterity). Error scoring using knowledge-based and skill-based domains	Significant increase in the total path length of the left hand across conditions, notably under mental and auditory stressors. Significant increase in the path length per movement of both hands across conditions, particularly under tactile stress and nearly under auditory stress. Errors significantly increased under all stress-inducing conditions. Skill-based errors increased notably under mental, tactile, and auditory stress, while knowledge-based errors increased across all conditions. Significant correlation between the total number of errors and the path length per movement of both hands	Not measured	Moderate ^2^	All three stressors led to impaired dexterity and an increase in the incidence of errors. Combined stressors had the greatest deterioration in performance

*Heart rate variability metrics: *HR* Heart rate, *HRV* heart rate variability, *R-Ri* R to R interval, *SDNN* Standard deviation of inter-beat intervals, *LF/HF* low frequency/high frequency ratio, *RMSSD* Root mean squared of the successive differences between normal heartbeats

Physiological variables: *MAP* mean arterial pressure, *BP* blood pressure, *f-NIRS* functional near-infrared spectroscopy, *NASA-TLX* National Aeronautics and Space Administration Task Load Scoring, *SURG-TLX* Surgical Task Load Scoring, *STAI* State-trait anxiety index, *STAI-6* Short form state-trait anxiety index

#### Technical skills

A single prospective observational study of high risk of bias demonstrated the effect of stress on technical skills. The study of 23 bariatric procedures, demonstrated a statistically significant higher error rate using the Generic Error Rating Tool in stressed time periods, compared to baseline heart rate variability in 1-, 2- and 5-min time periods [37]⁠. The study only involved a single surgeon resulting in the high degree of bias and thus limits the generalisability of these findings.

#### Non-technical skills

Three prospective observational studies of moderate risk of bias demonstrated a deterioration of non-technical skills in response to acute stress. Anton et al. demonstrated higher stress and workload resulting in lower non-technical skills amongst 15 surgeons [38]⁠. Overall, stress was associated with impaired situational awareness, decision making and the consideration, selection and communication of surgical options, irrespective of experience [38]⁠. In a general surgical environment, two experienced surgeons demonstrating lower stress (measured using physiological variables) were found to have higher task engagement [39]⁠. Sexton et al. demonstrated significantly higher anticipation ratio, higher non-verbal requests and longer request durations in three surgeons performing robotic prostatectomies when reporting stress [40]⁠. These findings are again limited by the large heterogeneity in the methods used to assess non-technical skills utilising both validated and non-validated criteria.

### Effect of acute stress on surgeons with patient outcomes

No studies were identified demonstrating that surgeon stress may have a causal relationship on patient outcome. Multiple studies were encountered during screening that compared outcomes for laparoscopic versus robotic surgery, however, none fulfilled the inclusion criteria as they did not directly examine a causal relationship between stress and performance.

## Discussion

From the available literature it can be seen that acute stress within the peri-operative period has a direct impact on surgical performance. In both simulated and live surgical environments, there is a negative effect of acute stress on both technical and non-technical skills.

Technical skill impairment from stress was well described in the included studies. Surgeon kinematics appear to have clear linear relationships with acute stress [28]⁠. Increased procedural time under stress, despite more rapid movements is explained by increased path lengths, reduction in economy and higher error rates. The adage of “slow is smooth and smooth is fast” certainly holds true when stress is high. Questions of the underlying mechanism of this movement inefficiency is yet to be understood. Whether this is due to direct effects of stress on visuomotor skills, cognitive processes or a combination of both is unclear. Further research could explore the degree to which the increase in movement inefficiency is caused by changes in movement planning or control.

Non-technical skills were also affected by acute stress. As non-technical skills require additional cognitive resources it is unsurprising that in the presence of stressors, performance in this domain deteriorates. Although Anton et al. demonstrated a general association with stress and non-technical skills regardless of experience, the literature suggests that experience may have a protective effect [8, 13]⁠. This may be explained by the reduction of cognitive load by caching of skills and procedural automaticity gained from experience, resulting in reduced overall workload thus freeing up cognitive resources to maintain performance [13, 41]⁠. Three studies investigated the use of mental practice with relation to performance under stress conditions [31, 32, 42]⁠. Louridas et al. did not demonstrate any difference in non-technical performance with mental practice although did observe improvement in technical skills [32]⁠. Two other studies reported improvements in mental practice groups, suggesting that stress reduction via mental practice improves performance which collaborates with findings from qualitative studies [12]⁠. Given the evidence for the significant impact of non-technical skills on surgical performance, it is surprising that only a minority of studies examined this domain [8]⁠.

Several factors were found to moderate the effect of acute stress on performance. Crewther et al. demonstrated that practice resulted in reduction in stress and subsequent improvement of performance [43]⁠. Stress coping strategies also appear to moderate the effect of stress on technical performance [31, 33]⁠. This finding supports those found in qualitative literature and supports a linear relationship between stress and performance deterioration [12]⁠. Further to deterioration of physical technical skills, cognitive load from stress may result in attempts to compensate for reduced cognitive resources, with cognitive lock-up, use of heuristics and simplification of decision making strategies used to compensate but thus resulting in further performance deterioration [8]⁠.

No studies were identified demonstrating the effect of acute stress on patient outcomes. Given that qualitative evidence suggests that acute stress has significant effects on surgeon performance this was somewhat surprising. Anton et al. demonstrated 40% of surgeon respondents directly linked surgeon stress with an adverse patient outcome [44]⁠. This would collaborate with the findings of a recent systematic review on burnout in surgeons which demonstrated a heightened risk of medical adverse events in clinicians reporting burnout [11]⁠. It is also well recognised that there is a positive relationship between technical skill and patient outcome [15]⁠. It is therefore concerning that despite previous reviews noting the outcomes of stress on surgical performance and the correlation between performance and patient outcomes not much has changed in over a decade [13]⁠. Although establishing a causal effect of acute stress directly impacting on patient outcomes is challenging given the multitude of factors involved, there is clearly a need for such enquiry.

This review has several limitations. Given the lack of clarity in terminology and the difficulty in defining stress, it is understandable that the literature is highly heterogeneous. A mixed population of healthcare professionals were included due to the sparsity of the literature amongst surgeons alone. The reporting of stress in both subjective and objective physiological measurements was also highly varied. Outcome measurements for both technical and non-technical skills were also highly variable although most utilised objective quantitative measurements. Only 11 of the 19 studies provided a specific exposure to stressful stimuli with the remainder measuring stress during surgical cases. Thus, a causal effect is challenging to ascertain, especially when associating errors to stress or vice versa. In interpreting the results, it is therefore crucial to acknowledge the significant heterogeneity across multiple domains, serving as the key limitation of this review.

Overall, these findings corroborate the qualitative evidence looking at the effects of acute stress on surgeons [12]⁠. With eleven of the included papers published within the last decade, there has been an increased focus on the effect of stress on surgical performance but there are still many unanswered questions.

One key question is how stress and performance can be reliably and objectively quantified, using methods applicable to real-world operating theatres, to allow measurement of acute stress within the operating environment. Psychophysiological metrics, including electroencephalography, eye-tracking, heart rate variability and surgeon kinematics, may be the key real-time objective measurement of stress [18]⁠. This may also address a key limitation when addressing causality between stress and outcome; allowing determination of the presence of stress prior an adverse event such as intra-operative bleeding or as a result of the event. Thus, validated objective measurements would not only improve the understanding of stress within surgery but other high-pressure environments as well.

Using validated objective measures to derive where the threshold between appropriate and excessive levels of stress for optimal performance would not only benefit surgical procedures and patients but also have beneficial effects in the training of surgeons and surgical teams. This review did not specifically examine the effect of stress in training scenarios. Several studies did however demonstrate benefit from strategies to manage and reduce the impact of stress [32, 42]⁠. This finding highlights the utility of applying stress to training in a safe simulated environments; a practice that is frequent in many other fields such as aviation and industry. Given the varying responses noted between experts and novices, there may also be a role for the operators response to stress to act as a marker for surgical skill and expertise [8, 12]⁠.

Utilising objective measurement would also allow research into the impact of the multiple factors contributing to stress within this environment. It is clear from the literature that a multi-modal approach is a more sensitive to multiple factors [45]⁠⁠. However, implementation is not without challenge, given that previous methods such as the Imperial Stress Assessment Tool have not been widely utilised [45]⁠. Improved objective quantitative measurement may also allow validation and refinements to currently utilised stress mitigation techniques; mental practice shown to not only improve performance but also retain skills without interim practice, these are yet to be widely utilised [31, 32, 42]⁠

Given the growing recognition of the effects of mental health conditions, an even larger question is the relationship between acute and chronic stress as well as its effect on surgical performance. This is challenging area to investigate given the multitude of factors that give rise to chronic stress and the very individual response to both internal and external stressors. The growing adoption of biometric devices such as smart watches and rings within the population may hold the key to understanding this relationship given their ability for longer-term non-invasive monitoring.

In summary, acute stress has a direct effect on both technical and non-technical skills of surgeons in both simulated and real-world environments. Further research is required to understand how to reliably measure stress in the real-world operating theatre and mitigation strategies to prevent stress reducing surgical performance.

## Supplementary Information

Below is the link to the electronic supplementary material.

Supplementary file1 (docx 5 KB)
