# Supplementary material for: The effects of stress on surgical performance: a systematic review
Source: Surg Endosc. 2024 Dec 3;39(1):77–98. doi: 10.1007/s00464-024-11389-3 (PMC11666721; doi:10.1007/s00464-024-11389-3)
Supplement: Supplementary file 1 — Supplementary file1 (docx 5 KB) [file 464_2024_11389_MOESM1_ESM.docx]

Search strategy

MEDLINE, Embase, Cochrane Database, Web of Science, PsyINFO:

1 "anxiety".mp.

2 "cognitive load".mp.

3 "stress".mp.

4 "surgeon".mp.

5 1 or 2 or 3

6 (5) adj7 surgeon).mp.

7 limit 6 to human

8 limit 7 to yr="1990 -Current"
